# Heterogeneous Fenton Reaction at Circumneutral pH Values Catalyzed by Novel One-Pot Synthesized Iron Oxide/Silica Core–Shell Nanoparticles

**DOI:** 10.1021/acsomega.6c06904

**Published:** 2026-10-01

**Authors:** Bastian Rödig, Daniel Nagin, Rainer Müller, Werner Kunz

**Affiliations:** Institut für Physikalische und Theoretische Chemie, Universität Regensburg, D-93053 Regensburg, Germany

## Abstract

The use of iron oxide catalysts in advanced oxidation processes (AOP), such as the conversion of hydrogen peroxide to reactive oxygen species (ROS), is relatively inefficient at circumneutral pH values. Silica-containing iron oxide nanoparticles derived from a one-pot process show higher conversion rates and more efficient utilization of ROS compared with those made of plain iron oxide. The as-formed particles (50–200 nm) were characterized by TEM, SEM, BET, and DLS and used in a reaction to degrade phenol. In a period of 8 h, all particles investigated showed higher phenol degradation while degrading significantly less hydrogen peroxide. The higher conversion rate can be attributed to the interaction of iron with the silica shell, which alters the redox processes and favors the formation of stronger oxidants during H_2_O_2_ decomposition. Production of such nanomaterials by a simple, environmentally friendly, and upscalable route is particularly appealing due to its simplicity in a one-pot process.

## Graphical abstract

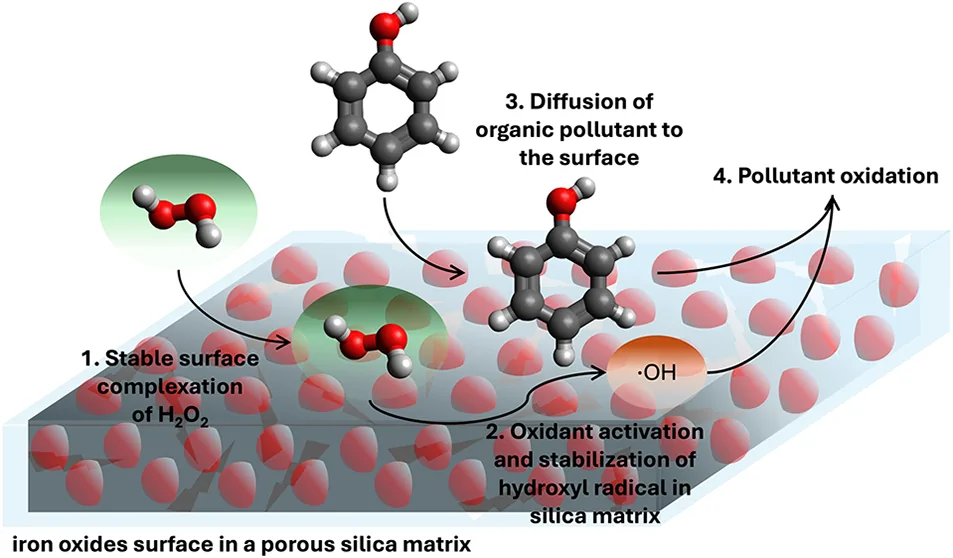

## Introduction

Iron is the fourth most common element in the Earth’s crust and occurs in various oxides/hydroxides.^1^ One of the possible applications is the use as a catalyst for advanced oxidation processes (AOPs) to oxidize pollutants in water, especially useful in the treatment of wastewater.^2,3^ AOPs are typically characterized by the hydroxyl radical (^•^OH), which bears a high standard reduction potential of *E* ^0^ = 2.80 V, which is even higher than that for atomic oxygen (*E* ^0^ = 2.42 V). One prominent example of AOPs is the so-called Fenton reaction. In this reaction, a soluble iron salt, most commonly iron­(II) sulfate, is the catalyst for the decomposition of the H_2_O_2_ at acidic pH values of 3 or less.^4^ The commonly accepted mechanism of the catalytic decomposition of H_2_O_2_ in the presence of solubilized Fe^2+^ has been described in the literature on the basis of the classical Haber–Weiss mechanism.^1,5^

1 $$H_{2}O_{2}+Fe^{2+}\to Fe^{3+}+OH^{-}+{HO}^{•}$$

Numerous different decomposition mechanisms initiated by Fe^3+^, such as the Weiss mechanism for decomposition of H_2_O_2_ on metal surfaces,^6^ have been proposed in the past, but cannot appropriately describe the mechanism over iron oxide surfaces. The more complex and more accurate descriptions can be found in ref 7. Reactions are classified into homogeneous and heterogeneous Fenton­(-like) reactions for soluble and insoluble compounds, such as iron sulfate and iron (hydr)­oxides, respectively. The heterogeneous reaction can overcome several drawbacks of the homogeneous reaction and also has its own problems.^1,8^ For the heterogeneous Fenton­(-like) reactions, various properties of the catalysts are important, such as the oxidation state of the iron,^9^ the crystallinity,^10,11^ and the surface area.^11^ Also, the doping with other transition metals or the implementation of the iron oxide in secondary structures is possible and can improve the catalytic ability. The synthesis of such particles usually includes multiple steps.^9,12^

In this work, variations of a simple one-pot approach are used for the synthesis of silica-coated iron sulfide/carbonate nanoparticles.^13,14^ Here, the coupling of a pH-active anion with the polycondensation of solubilized silica species results in coprecipitation of nanoparticles.^13,15–17^

The as-formed particles can then be further processed into iron oxides by a reaction with atmospheric oxygen at elevated pH values.^18^

For the iron sulfides, the reaction with the oxides occurs via the hydroxides. Depending on the starting oxidation state, a further oxidation takes place before the condensation of the hydroxides results in the oxides.^18^ This reaction results in mostly amorphous iron oxide covered in a silica shell, which increases the total surface area and the colloidal stability and allows size control of the primary iron oxide particles.^14^ The optimal synthesis parameters for this application are determined by variation of the pH, silicate concentration, addition of small molecules, change of iron precursors (Fe^2+^ or Fe^3+^), and variation of primary precipitated particles.

The heterogeneous Fenton reaction was employed as a proof-of-concept application for the synthesized particles. Because of its strong dependence on the accessibility of surface iron sites, this reaction provides a sensitive tool to evaluate whether the one-pot synthesized silica-coated iron oxide particles present catalytic functionality. In addition, the catalytic performance can be quantified by monitoring the hydrogen peroxide decomposition and phenol degradation kinetics. As a technologically relevant process in wastewater treatment, the heterogeneous Fenton reaction therefore represents a suitable model system for assessing the applicability of synthesized materials.

## Results and Discussion

The optimal synthesis conditions for obtaining catalytically active particles via the one-pot approach^13,14^ have been systematically investigated. First, the structure of the particles was investigated, and then their ability as a catalyst in a Fenton reaction at circumneutral pH values was evaluated. Typically, the structures formed in this coupled precipitation process result in small primary particles embedded in diffuse silica matrices forming the secondary particles (cf. Figure 1a).^14^

**Figure 1:**
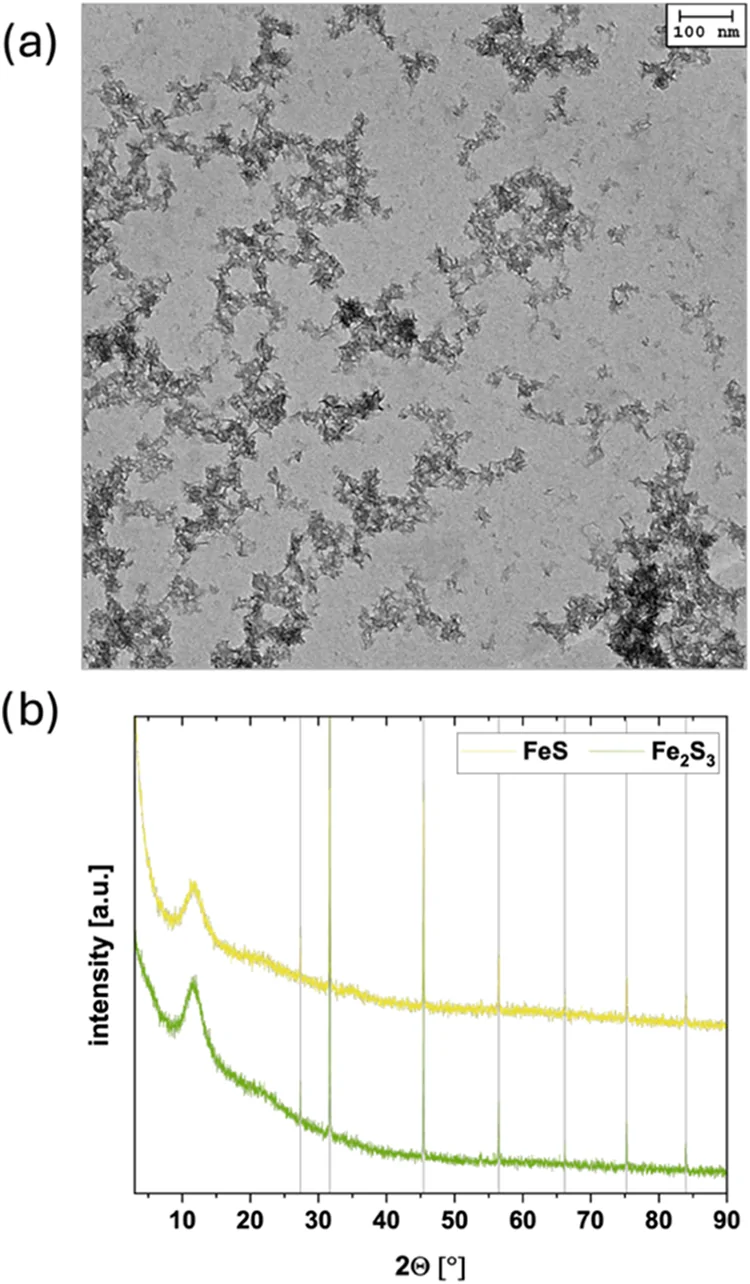
(a) TEM micrograph of particles derived from Fe­(II) at 5 mM TEOS and a pH value of 11. The scale bar corresponds to 100 nm. (b) XRD spectra of iron sulfide derived from Fe­(II) and Fe­(III), respectively. The vertical lines indicate the NaCl crystal structure as a reference.

While particles derived from the same iron precursor appear quite similar, rather different structures occur when different precursors are used (Figure S1). An increase in the Fe/Si ratio results in smaller secondary particles in the observed range. Particles derived from Fe­(III) are significantly smaller than those derived from Fe­(II). Hydrodynamic radii derived from DLS for selected particles are summarized in the Supporting Information (Table S1). Generally, the hydrodynamic radius decreases with increased silica concentration or increasing pH during synthesis due to increased colloidal stability in the early stages of particle formation.^14^

No representative reflexes for iron oxides could be found in XRD measurements, but a broad peak at small angles, which indicates the existence of amorphous crystal structures (cf. Figure 1b). The visible reflexes derive from the synthesis residue NaCl. It should be noted that the absence of reflexes can also be due to the too small sizes of the primary particles (<5 nm). Neither the XRD nor the TEM provides information about the crystal structure of the formed iron oxides, except that a certain proportion may be amorphous. Amorphous crystal structures are potentially of particular interest for catalytic applications due to the repeatedly reported increased reaction rates compared to the crystalline counterparts.^19–22^ Nevertheless, in future studies, the exact composition of the material and the distribution of iron oxidation states should be examined using complementary techniques such as Mössbauer spectroscopy, Raman spectroscopy, or X-ray photoelectron spectroscopy (XPS). The latter may be particularly informative given the surface-sensitive nature of the heterogeneous Fenton reaction, as it would allow the Fe­(II)/Fe­(III) ratio to be probed specifically at the particle surface, i.e., at the sites accessible to H_2_O_2_.

Synthesizing iron carbonates first with calcination after lyophilization results in more pronounced crystal structures compared to FeS-derived particles. While the XRD spectrum of the precipitated iron carbonate shows no reflections apart from a slight indication of amorphous structures, several reflections can be seen after calcination, which indicates various iron (hydr)­oxides (cf. Figure S2). During the calcination/sintering process, the particles rearrange into a classical lattice.^23,24^

Because of the chosen calcination temperature (750°C), the main component should be α-Fe_2_O_3_, also known as hematite,^25^ which is frequently used in the literature as a highly reactive heterogeneous Fenton catalyst.^26^

The indication for amorphous structures vanishes for the particles derived from Fe­(III) species but remains for the Fe­(II) derived particles. The reflexes in both spectra can be attributed to hematite and magnetite (Fe_3_O_4_). TEM micrographs of iron carbonates and calcinated samples can be found in the Supporting Information (Figure S3).

Particles derived from both pathways, oxidation of FeS (cf. Figure 2a) and the calcination of FeCO_3_ (cf. Figure 2b), were used as catalysts for phenol degradation in a heterogeneous Fenton reaction. To this purpose, a series of particles were produced at different pH values and with different amounts of silica. Hematite was used as a reference and consistently resulted in a total of ∼14% phenol decomposition after 8 h. Only a few particles derived from FeCO_3_ exhibit poorer conversion rates, whereas this is not the case for particles derived from FeS.

**Figure 2:**
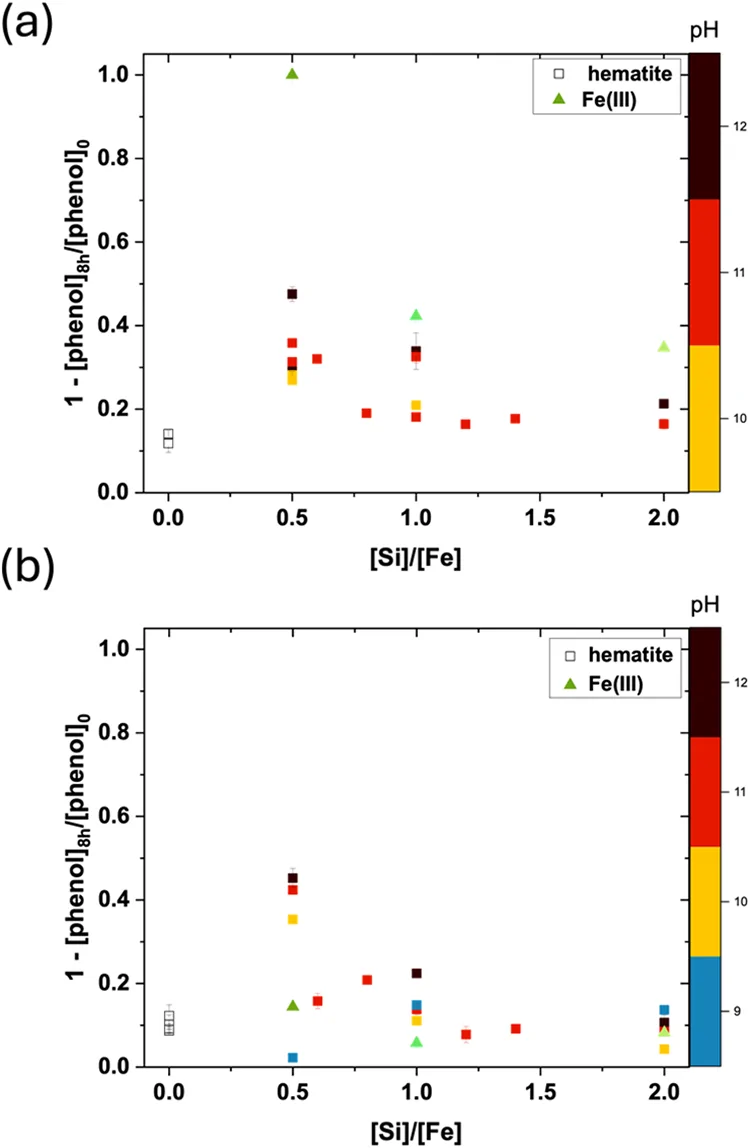
Relative amount of phenol degraded after 8 h reaction time for particles derived from modified syntheses. The white squares represent the reference hematite. Panel (a) shows particles derived from FeS, and panel (b) shows particles derived from FeCO_3_. The green triangles represent particles derived from Fe­(III) at the best conditions found for Fe­(II). The colorscale represents the synthesis pH.

A trend across all particles is the reduction in phenol decomposition with increasing silica content (cf. Figure 2). This can be directly attributed to the surfaces of the catalytically active cores being better shielded. In other words, the density and thickness of the silica shell increase, thereby preventing more radical release reactions on the iron oxide surfaces.^14^

Another trend is the increased level of phenol degradation for particles originating from syntheses at higher pH values. This applies to both particles originating from FeS and those originating from FeCO_3_. Because of chemical equilibria, the synthesis of FeCO_3_ can be carried out at a lower pH value than FeS.^14^ Only at pH 12 and higher silica concentrations, this trend is no longer strictly true.

Directly compared, particles derived from FeS exhibit a higher phenol degradation. While the difference is small at low silica content, it increases significantly toward a higher silica content. At an Si/Fe ratio of 1, twice as much phenol is converted using FeS-derived particles as with particles derived from FeCO_3_. Even more evident is the difference between Fe­(III)- and Fe­(II)-derived particles. While for FeS the change in the iron precursor leads to particles with an increased phenol degradation capacity, particles obtained from FeCO_3_ exhibit poorer catalytic properties (cf. Figure 2).

On the basis of these results, no further experiments were conducted on particles derived from FeCO_3_, as particles derived from FeS appeared to be more promising. In addition, the preparation of FeS-derived particles is less time-consuming. The Si/Fe ratio and the specific surface area of selected lyophilized particles determined by BET are listed in Table 1.

**Table 1:** Surface Area per Gram of Nanoparticles Measured with BET^a^

Si/Fe ratio	A(Fe^(II)^) [m^2^/g]	A(Fe^(III)^) [m^2^/g]
0.5	296 ± 11	274 ± 12
1	318 ± 3	231 ± 12
2	332 ± 5	204 ± 4

a Particles derived from an iron sulfide synthesis at different silicate concentrations.

The BET measurements show that the synthesis with Fe­(II) always results in a higher surface area, regardless of the silica concentration used for the synthesis. Also, the surface area increases for increased silica concentrations, whereas for Fe­(III), the surface area decreases with increased silica concentrations. This seems to be a direct result of classical nucleation theory and the increased insolubility of Fe­(III) sulfides over Fe­(II) sulfides (cf. Table S2) and corresponds to the sizes determined by DLS (cf. Table S1).

Faster precipitation results in more primary particles and a less diffuse silica matrix, which induces the majority of the measurable surface for BET. Also, the size of the particles derived from Fe­(III) does not change significantly, indicating increased tightness of the shell due to closure of inner surfaces with the addition of more silica during synthesis.^27^ Evaluation of SEM (cf. Figure S4) highlights the porous surface of the material and the aforementioned trend. EDX measurements indicate that the actual Si/Fe ratios for the particles are reduced to about two-thirds compared to the theoretical value (cf. Table S3).

When the particles are used as catalysts in Fenton reactions at circumneutral pH, a few trends emerge. First, all particles used induce higher phenol degradation after 8 h compared to the hematite used as a reference.^12^ Second, all particles derived from Fe­(III) cause higher phenol degradation than any particle derived from Fe­(II). Third, the phenol degradation is increased for particles with lower Si/Fe ratios compared to higher ratios (cf. Figure 3a).

**Figure 3:**
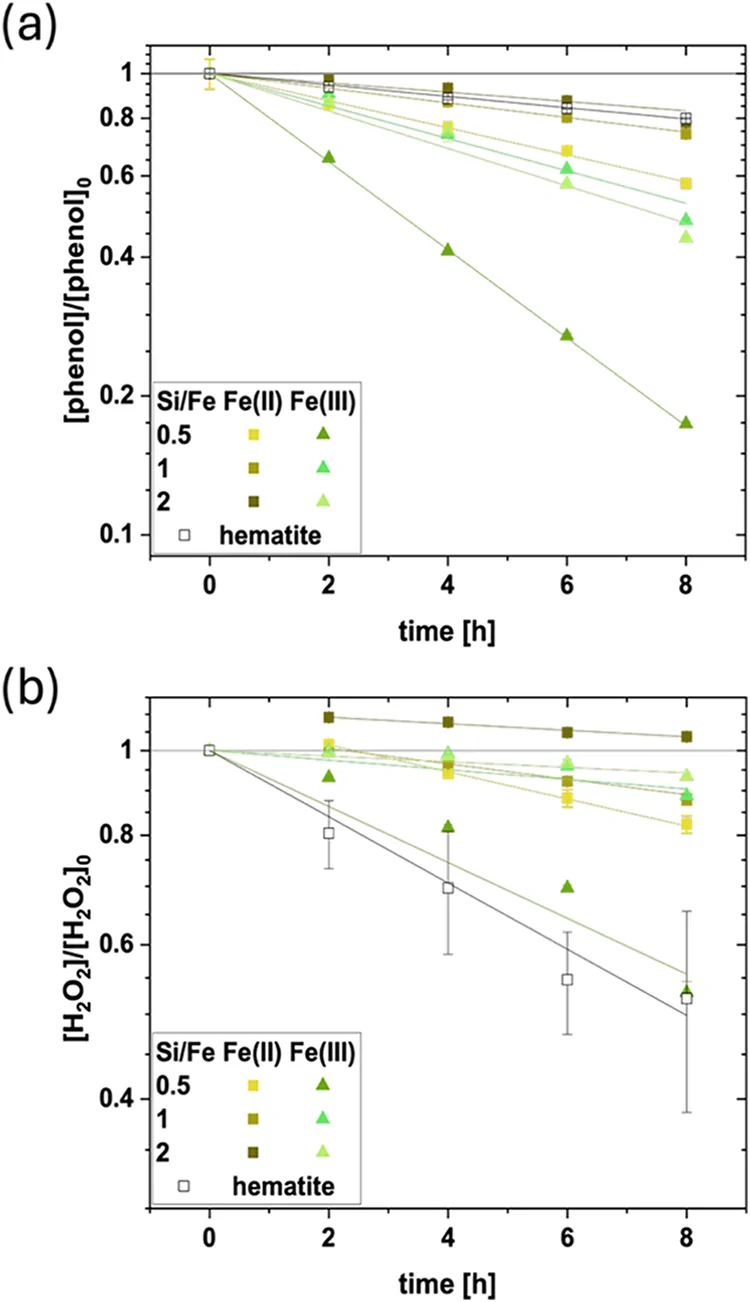
(a) Normalized degradation of phenol over the reaction time of 8 h for different particles derived from Fe­(II) or Fe­(III) and the reference, hematite. (b) Normalized degradation of H_2_O_2_ over the reaction time of 8 h for different particles derived from Fe­(II) or Fe­(III) and the reference, hematite. Note that the pH value during the reactions was always between 5.6 and 5.3.

Similar and different trends can be observed in the degradation of H_2_O_2_. Here, the reference hematite causes the highest rate of H_2_O_2_ degradation. A higher Si/Fe ratio again leads to less reactant conversion, with Fe­(III) at Si/Fe = 0.5 reducing the H_2_O_2_ concentration similar to the reference hematite. All other particles cause a smaller H_2_O_2_ reduction with a similar course over a period of 8 h (cf. Figure 3b).

Another key factor for the reaction rates in Fenton reactions is the pH value. In this case, the pH was above 5.3 for all samples (cf. Figure S5), in a circumneutral range, where the radical pathway for H_2_O_2_ decomposition is already less favored.^7^

The overall surface area of the particles after lyophilization increases with increasing silica concentration for synthesis derived from Fe­(II) but decreases for Fe­(II). Notably, the trends in BET surface area do not mirror the trends in catalytic activity (cf. Figure 3a), suggesting that accessible iron site density, rather than total surface area, is the limiting factor for catalytic performance (cf. Table 1).

At the highest Si/Fe ratio (2), the cores are effectively shielded as evidenced by their poor catalytic efficiency. At lower ratios of 1 or 0.5, the particles showed catalytic H_2_O_2_ decomposition. The leakage or dissolution of iron is almost negligible and does not significantly increase for lower Si/Fe ratios. After 8 h, only a small percentage (<0.5%) of the total iron used was dissolved into the reaction medium (cf. Figure S6). This also proves that even though negligible amounts of iron are in solution, the majority of the catalyst remains insoluble, and the catalysis remains heterogeneous. This ensured easy removal of the catalyst after the reaction. While these results indicate good short-term stability under the applied conditions, a systematic assessment of long-term stability, including catalyst reuse over multiple cycles and structural characterization (e.g., by SEM and XRD) of the material after reaction, was beyond the scope of this study and remains an important direction for future work.

When the degradation of phenol and H_2_O_2_ is compared directly, a pattern becomes apparent (cf. Figure 4). Even though all particles used induced less H_2_O_2_ degradation than hematite, the phenol degradation was higher compared to the reference hematite. In fact, all particles derived from Fe­(III) induce a higher phenol degradation with less H_2_O_2_ used compared to that of Fe­(II).

**Figure 4:**
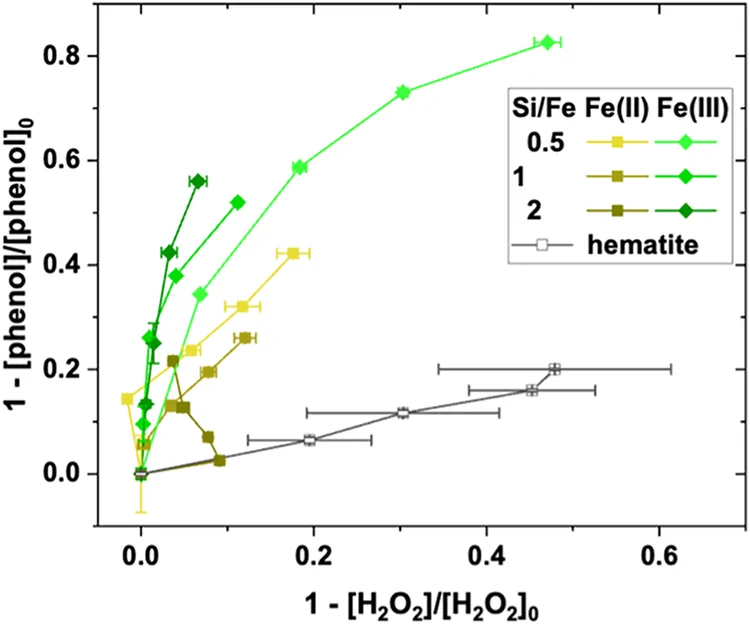
Normalized degradation of phenol with respect to the normalized degradation of H_2_O_2_ for different particles derived from Fe­(II) or Fe­(III) at different Si/Fe ratios and the reference, hematite.

The degradation of the peroxide is much more controlled, and the reactive oxygen species (ROS) are harnessed much more efficiently on the silica surfaces.

Numerous studies reported significantly enhanced oxidant utilization efficiency for silica-supported iron oxides compared to unsupported iron oxides. As the underlying mechanism remains controversial,^12,19,28,29^ the following mechanistic considerations should be regarded as a hypothesis, as a detailed mechanistic investigation was beyond the scope of the present study.

For example, silica- and alumina-containing iron oxides were reported to exhibit a stoichiometric efficiency (mol of pollutant transformed per mol of H_2_O_2_ consumed) 10 to 80 times higher than hematite, goethite, or amorphous FeOOH for phenol degradation at circumneutral pH,^12^ a comparison directly relatable to our own hematite-referenced results. For a broader overview of near-neutral-pH heterogeneous Fenton catalysts, we refer the reader to recent reviews.^28,29^

One explanation for the enhanced performance is the increased dispersion of the iron centers. Silica inhibits aggregation and reduces the particle size, resulting in a higher density of accessible surface iron sites. Consequently, a larger fraction of iron atoms participates in the hydrogen peroxide activation compared with bulk iron oxides.^30,31^

Silica may also directly influence the interfacial reactions. Silanol groups near the iron surface site participate in hydrogen bonding with adsorbed peroxides and reactive iron species. This may delay desorption kinetics and facilitate charge-transfer processes at the oxide–water interface. Additionally, silica surfaces exhibit lower reactivity toward radical scavenging, thereby reducing unproductive side reactions and increasing oxidant utilization.^32–34^ It was also demonstrated that in this way, the diffusion of the pollutant to the surface-bound ROS can become the rate-determining step for the oxidation reaction.^19^

This behavior may stem from multiple reasons, such as hydrogen bonding with the siloxane network^35^ or potential negative charge transfer from the silica onto the iron oxide surfaces.^36^ To elucidate the exact mechanism, additional experiments, e.g., EPR/spin trapping or radical scavenger experiments, would be necessary.

## Conclusion

Particles derived from a simple one–pot synthesis have been successfully used as a catalyst in Fenton reactions. Similar to particles derived from more elaborate multistep processes,^12^ the particles show higher phenol conversion at comparatively high pH values compared to typical iron oxides such as hematite. The most relevant parameter for the synthesis and the catalytic ability is the Si/Fe ratio. First, the colloidal stability after synthesis increases with increasing silica concentration. Also, the overall size of the formed secondary structures decreases, and the size of the primary particles decreases.^14^ In addition, the shell thickness around the catalytically active cores increases, which results in decreased decomposition of H_2_O_2_ and phenol. Nevertheless, the silica shell remains a key factor for higher turnover rates and efficient harnessing of the reactive oxygen species (ROS). The balance between colloidal stability and shell permeability determines the efficiency of the catalyst. The mechanistic explanations proposed here for the enhanced ROS utilization remain hypothetical and should be corroborated in future work, for example, through EPR/spin-trapping or radical scavenger experiments. This one-pot process allows a simple synthesis of particles with good catalytic activity at elevated pH values, offering a high potential for wastewater treatment. Given its simplicity, scalability, and cost-effectiveness, this one-pot route is particularly attractive for decentralized water treatment units, and the heterogeneous, easily separable nature of the catalyst makes it a promising candidate for integration into continuous-flow systems for industrial wastewater remediation. Preliminary trials confirm that the process scales readily in reaction volume; extending this simplicity to higher precursor concentrations for more concentrated, industrially relevant batches is a natural next step for future work.

## Materials and Methods

Silica-coated iron sulfide and iron carbonate nanoparticles were synthesized by one-pot precipitation. An aqueous iron­(II) or iron­(III) chloride solution was added rapidly under vigorous stirring at room temperature to an aqueous solution containing pre-hydrolyzed tetraethyl orthosilicate (5–20 mM) and sodium sulfide or sodium carbonate (10 mM) at varying pH. After centrifugation, washing, and lyophilization, iron carbonate-derived particles were converted into oxides by calcination at 650 °C, and iron sulfide-derived particles by prolonged air oxidation. Particles were characterized by XRD, TEM/SEM, DLS, and nitrogen adsorption (BET). For the heterogeneous Fenton reaction, particles (3 mg/mL) were combined with phenol (0.5 mM) and H_2_O_2_ (50 mM) in citric acid buffer (pH 5.3) at room temperature. Phenol and H_2_O_2_ concentrations were monitored over 8 h by HPLC and the titanium sulfate method, respectively; leached iron was quantified via the 1,10-phenanthroline method. Note that full experimental details, including reagent sources, instrument parameters, and data evaluation, are provided in the Supporting Information.

## Supplementary Material



## Data Availability

Data for this article are available at Publikationsserver der Uni Regensburg at: https://epub.uni-regensburg.de/79067/1/Data.zip. Experimental data can be found in the Supporting information.

## Notes

Detailed Experiemental; additional TEM and SEM micrographs; Hydrodynamic radii (*r*_H_) derived from DLS measurements of iron oxide particles derived from Fe­(II) and Fe (III), respectively, at different Si/Fe ratios; XRD FeCO_3_; pH values of the reaction solutions over the 8 h reaction time; theoretical Si/Fe ratio and calculated mean values Si/Fe derived from EDX measurements from multiple SEM pictures; leached iron concentration after 8 h of reaction time (PDF)
