# Supplementary material for: Heterogeneous Fenton Reaction at Circumneutral pH Values Catalyzed by Novel One-Pot Synthesized Iron Oxide/Silica Core–Shell Nanoparticles
Source: ACS Omega. 2026 Oct 1;11(40):61496–501. doi: 10.1021/acsomega.6c06904 (PMC13670825; doi:10.1021/acsomega.6c06904)
Supplement: Supplementary file 1 [file ao6c06904_si_001.pdf]

SI - Heterogeneous Fenton reaction at circumneutral pH values catalyzed by novel one–pot synthesized iron oxide/silica core shell nanoparticles

Bastian Rödig[1*], Daniel Nagin[1], Rainer Müller[1], Werner Kunz[1]

[1] Institut für Physikalische und Theoretische Chemie, Universität Regensburg, D-93053 Regensburg, Germany.

Experimental

Chemicals:

The following substances were used as received without further purification: Ammonia solution 25% (NH_4OH, Supelco, >99%), citric acid monohydrate ($C_6H_8O_7$; Merck, >99.5), hydrochloric acid (1M HCl in water; Sigma-Aldrich, analytical grade), hydrogen peroxide (35% in water; Sigma-Aldrich), iron(II) chloride tetrahydrate ($FeCl_2 \cdot 4H_2O$; Sigma-Aldrich, ≥99%), iron(III) chloride hexahydrate ($FeCl_3 \cdot 6H_2O$; Carl Roth, >99%), methanol (CH_3OH; Sigma-Aldrich, >99.8%), 1,10-phenantroline ($C_{12}H_8N_2$, Sigma–Aldrich, ≥99%), phenol (Sigma-Aldrich, >99.9%), sodium carbonate (Na_2CO_3; Sigma-Aldrich, ≥99.8 %), sodium chloride (NaCl; Carl Roth, ≥99.8 %), sodium hexametaphosphate ($Na_6[(PO_3)_6]$, Fluka, >99.8%), sodium hydroxide (NaOH; Sigma-Aldrich, ≥98 %), sodium metasilicate (Na_2SiO_3; Sigma-Aldrich, >97%), sodium sulfide trihydrate ($Na_2S \cdot 3H_2O$; VWR, >60%), sulfuric acid (0.5M in water; Supelco, >95%), tetraethylorthosilicate ($Si[C_2H_5O]_4$, Sigma–Aldrich, >98%), trifluoroacetic acid (CF_3COOH; Sigma-Aldrich, >99%), and titanium(IV) oxysulfate solution (15% in water, Sigma-Aldrich, >99%). All solutions and dilutions were prepared using water taken from a Millipore system.

Synthesis of particles:

To avoid potential covalent attachment to the surface of glass vessels by silica condensation reactions, synthesis was carried out using plastic beakers. To 50 mL aqueous solutions containing varying amounts (5-20 mM) pre-hydrolyzed TEOS and 10 mM sodium sulfide or sodium carbonate the same volume of an aqueous solution of 10 mM iron chloride (Fe^{2+} or Fe^{3+}) was quickly added under vigorous stirring (700 rpm) at room temperature. Immediately after mixing, black (iron sulfide) or greenish (iron carbonate) suspensions of more or less finely dispersed precipitates were obtained. In some variations the pH of the silica containing solution is adapted to 9-12. Afterwards, the resulting particles were separated from synthesis residues by centrifugation in a Sigma 3-18KS centrifuge at 14000 rpm (20379 g) for 5 min at room temperature. After repeated washing with water they were redispersed using an ultrasonic probe UP200Ht from Hielscher and the corresponding dispersions were lyophilized to obtain a dry powder. Given the low solubility products of the relevant iron phases, precipitation of iron from solution is expected to be essentially quantitative; isolated yields after centrifugation and washing were not systematically determined in this study. To convert the as-formed silica-coated iron carbonates into oxides, they were calcined in a Thermicon T oven from Heraeus under atmospheric pressure at 650 °C for at least 6h. To convert the iron sulfides into oxides they were stirred for at least 48h to react with oxygen from the air before their work up.

Characterization of nanoparticles:
X-ray diffraction analysis (XRD) was carried out using a STADI-P device from STOE & Cie GmbH (Darmstadt, Germany), with CuK$_{\alpha 1}$ radiation (λ=1.540598Å). The samples were ground to form a homogeneous powder and scanned from 2° to 92° in 0.015° steps. Raw data were processed with the WinXPOW software package (Stoe & Cie) Powder X-ray diffraction patterns were compared with reference data from the Powder Diffraction File (PDF) database of the International Centre for Diffraction Data (ICDD). For dynamic light scattering (DLS) measurements, a CGS-3 Compact Goniometer System from ALV (Langen, Germany) combined with an ALV/LSE-5004 Multiple Tau Digital Correlator and a vertically polarized 22 mW HeNe laser (λ = 632.9 nm), was used. Suspensions as obtained after synthesis were filtered using a 450 µm CA-membrane and measured at a scattering angle of 90° with a constant temperature of 25 °C for 300 s. Using the 2nd order cumulant analysis [25,26] of the ALV-7004 Correlator software, the hydrodynamic radius and size distributions can be calculated from the autocorrelation function. Also, the CONTIN algorithm [27,28] was used to gather information from more noisy measurements.
The structure and size of the particles were evaluated using transmission electron microscopy (TEM) using a CM-12 transmission electron microscope from FEI/Phillips (Dreieich, Germany) with a tungsten cathode and 120 kV acceleration voltage. For this purpose, 10 µL diluted (usually 1:20) sample was placed on carbon-coated copper grids from Plano GmBH (S160) hydrophilized by treatment in a PDC-3XG plasma cleaner from Harrick Plasma. After soaking for 30s excess liquid was removed by blotting with a filter paper and the grids were left on another filter paper drying for 24h. Pictures were taken with the digital camera 1kx1k from TVIPS (Gauting, Germany). The images were processed and evaluated with the software EMMenu4.
The specific surface areas of selected, lyophilized particles were determined by nitrogen adsorption following the Brunauer-Emmett-Teller (BET) adsorption model using an Accelerated Surface Area and Porosimetry System ASAP 2010 of Micromeritics (Norcross, GA, USA).
Field emission scanning electron microscopy (SEM) images were recorded on a Phenom Pro-X scanning electron microscope (Phenom-World BV, Eindhoven, Netherlands). Particles were sputtered with Au/Pd for 30 s (2.45 kV, 22 mA) under an Argon atmosphere using a Polaron E 510 Series II Sputter Coater (Polaron Instruments, London, UK).
The specific surface areas of selected, lyophylized particles were determined by nitrogen adsorption following the Brunauer-Emmett- Teller (BET) adsorption model using an Accelerated Surface Area and Porosimetry System ASAP 2010 of Micromeritics (Norcross, GA, USA).

Oxidation of Phenol*:*
All reactions were carried out at room temperature in 20 mL reaction medium with open reaction vessels. Reactions were buffered with 1 mM citric acid at pH 5.3 adjusted using 1M NaOH or 0.5 M H_2SO_4. The initial phenol concentration was 0.5 mM and a catalyst concentration of 3 mg/mL FeO was used. The reaction started with the addition of H_2O_2 solution resulting in a total H_2O_2 concentration of 50 mM. Experiments were carried out at least in triplicate, and average values and deviations are presented in this work.

Analysis:
Every 2h for 8h 2 mL were deducted from the reaction vessel, filtered using a 0.22 µm nylon filter, and transferred into an HPLC vial. 100 µL of methanol were added to the

vial to quench radicals. Samples were measured in a high-performance liquid chromatography (HPLC) using a Waters Alliance HPLC system equipped with a 4.6 mm x 250 mm Altmann Analytik C18 5 µm column. A flow rate of 0.8 mL/min was set with a mobile phase consisting of 49.5% water, 49.5% methanol, and 1% trifluoroacetic acid.

H_2O_2 was determined by the titanium sulfate method [29] using the absorption of UV/Vis light (at 402 nm) by the formed titanium complex measured using U-1000 spectrophotometer from Hitatchi (Tokyo, Japan) after dilution with water to suitable optical densities in cells of 10 mm path length.

Phenol and H_2O_2 conversion were calculated as $(C_0 - C_t)/C_0 \times 100$ %, where C_0 and C_t denote the initial concentration and the concentration at time t, respectively, based on external calibration curves recorded under identical measurement conditions.

Leeched iron in the solution during the reaction was measured using 1,10-phenanthroline as a complexing agent. [30] Using the absorption (at 510 nm) by the formed iron complex measured using U-1000 spectrophotometer from Hitatchi (Tokyo, Japan) after dilution with water to suitable optical densities in cells of 10 mm path length.

Supporting Information

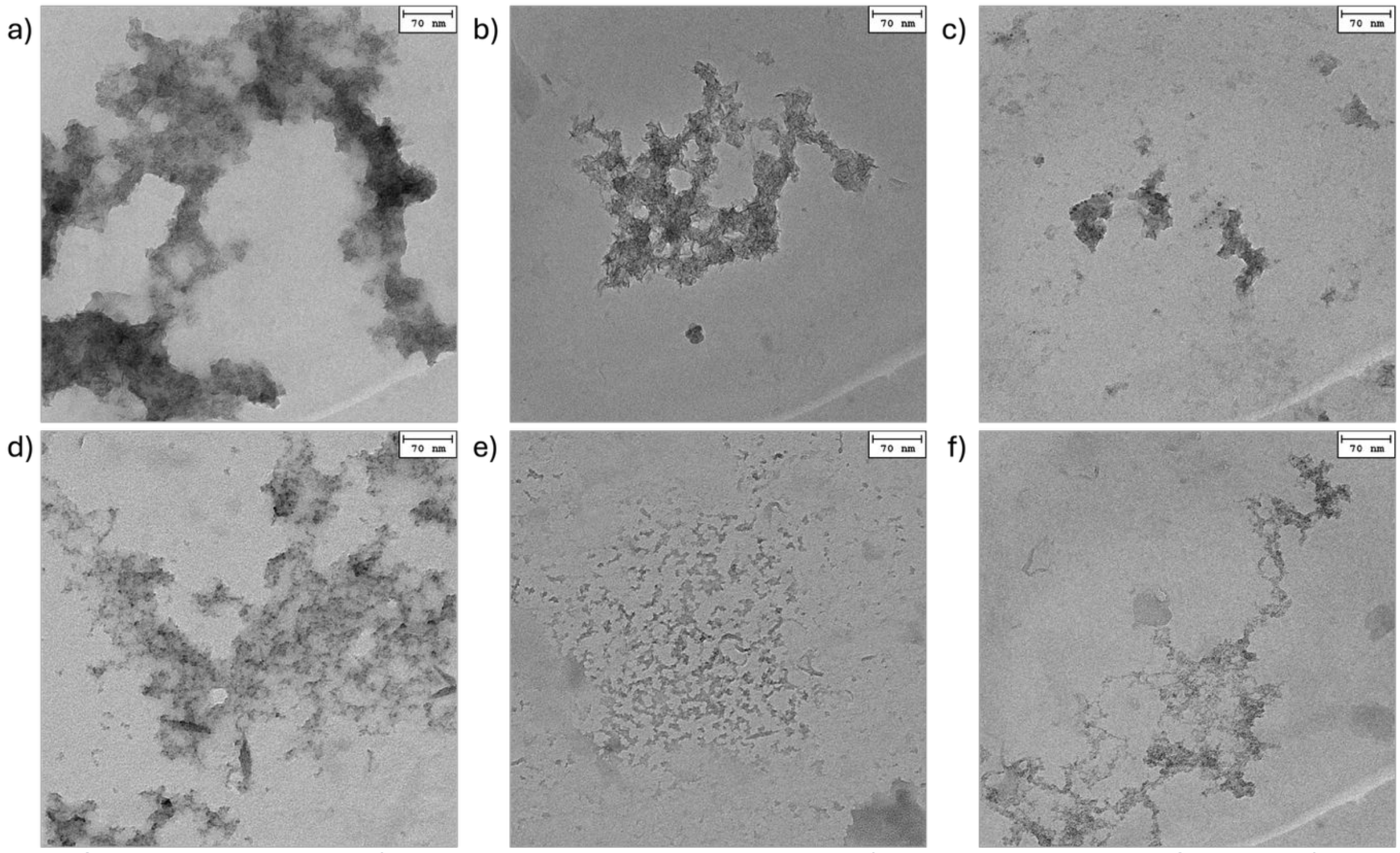

Figure S1. TEM micrographs of various iron oxides (a-c) Fe(II) and (d-f) Fe(III) with increasing Si/Fe ratio from left to right (a&d) 0.5, (b&e) 1, and (c&f) 2. The scale bar corresponds to 70 nm.

Table S1. Hydrodynamic radii (r_H) derived from DLS measurements of iron oxide particles derived from Fe(II) and Fe(III), respectively at different Si/Fe ratios. The pH value for synthesis was 11.

Si/Fe	Fe(II)		Fe(III)	
	size	PD.I.	size	PD.I.
0.5	196.74 ± 111.08	0.319	83.82 ± 48.36	0.333
1	113.86 ± 61.93	0.296	83.03 ± 49.68	0.358
2	68.09 ± 33.08	0.236	74.38 ± 45.2	0.369

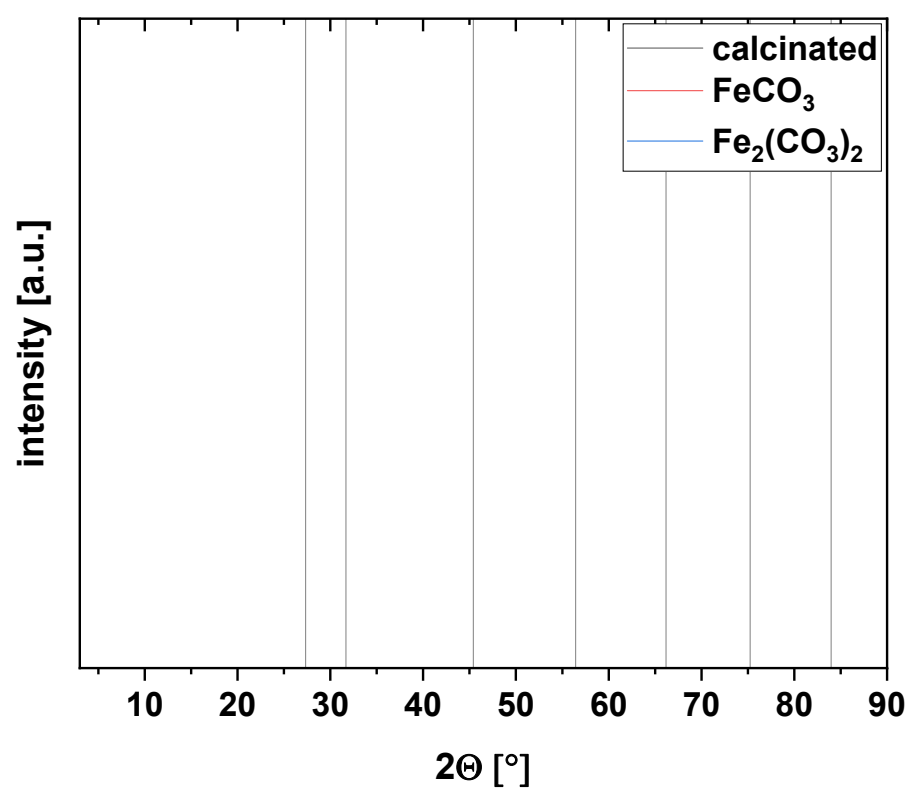

Figure S2. XRD FeCO3

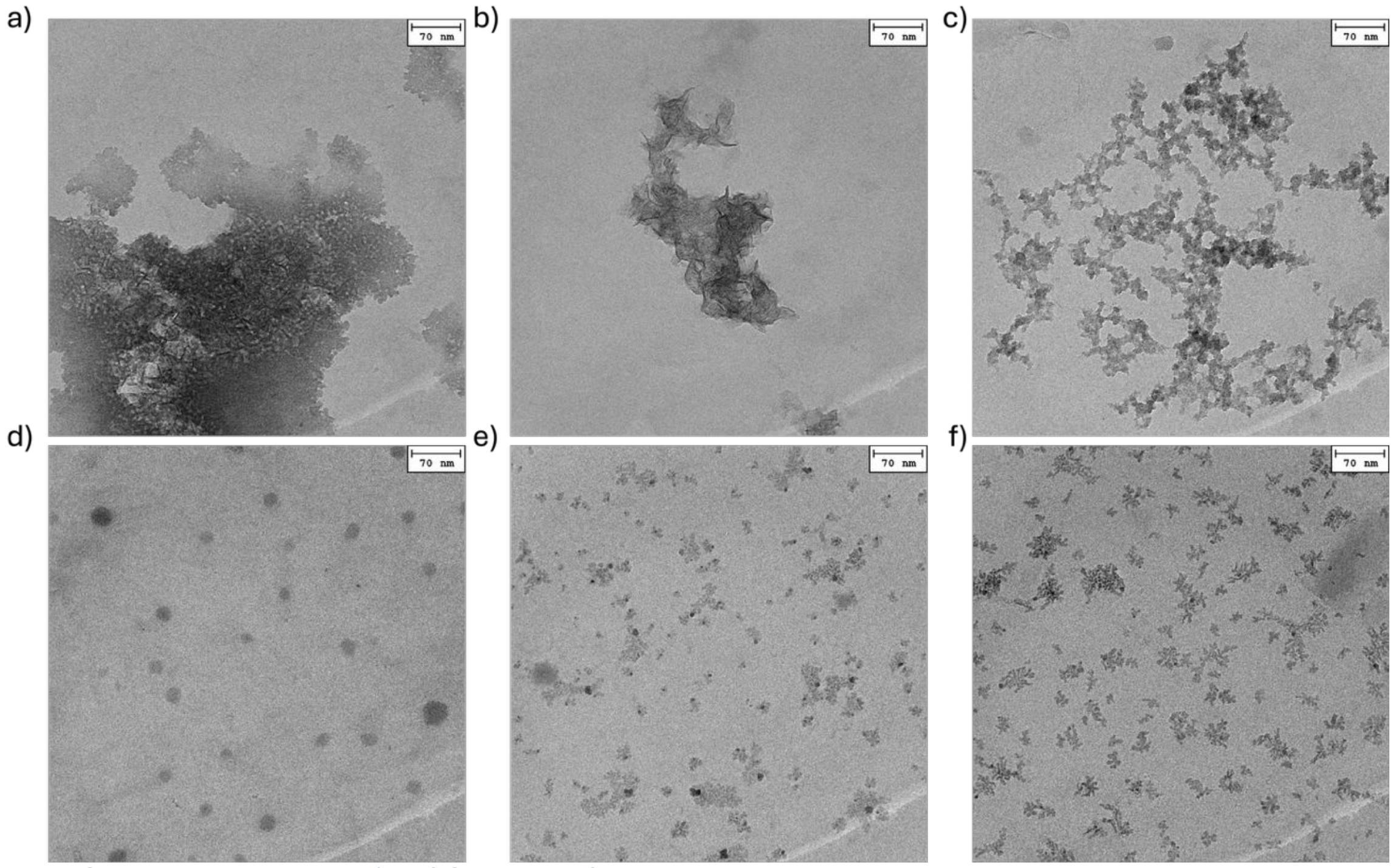

Figure S3. TEM micrograph of FeCO3 and FeO 2.5 und 5 und 5 Fe(III)

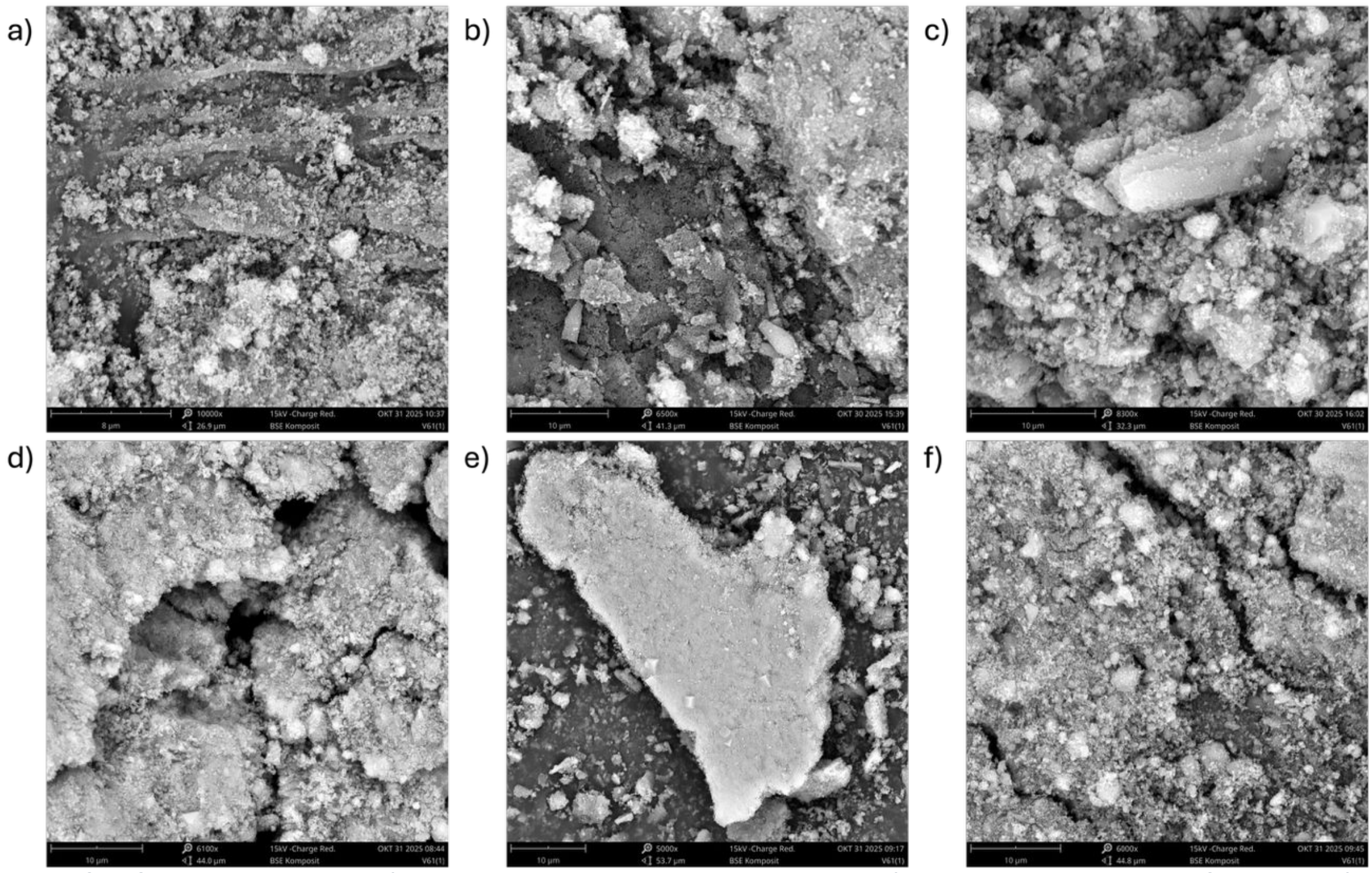

Figure S4. SEM micrographs of various iron oxides (a-c) Fe(II) and (d-f) Fe(III) with increasing Si/Fe ratio from left to right (a&d) 0.5, (b&e) 1, and (c&f) 2. The scale bar corresponds to (a) 8 µm and (b-f) 10 µm, respectively.

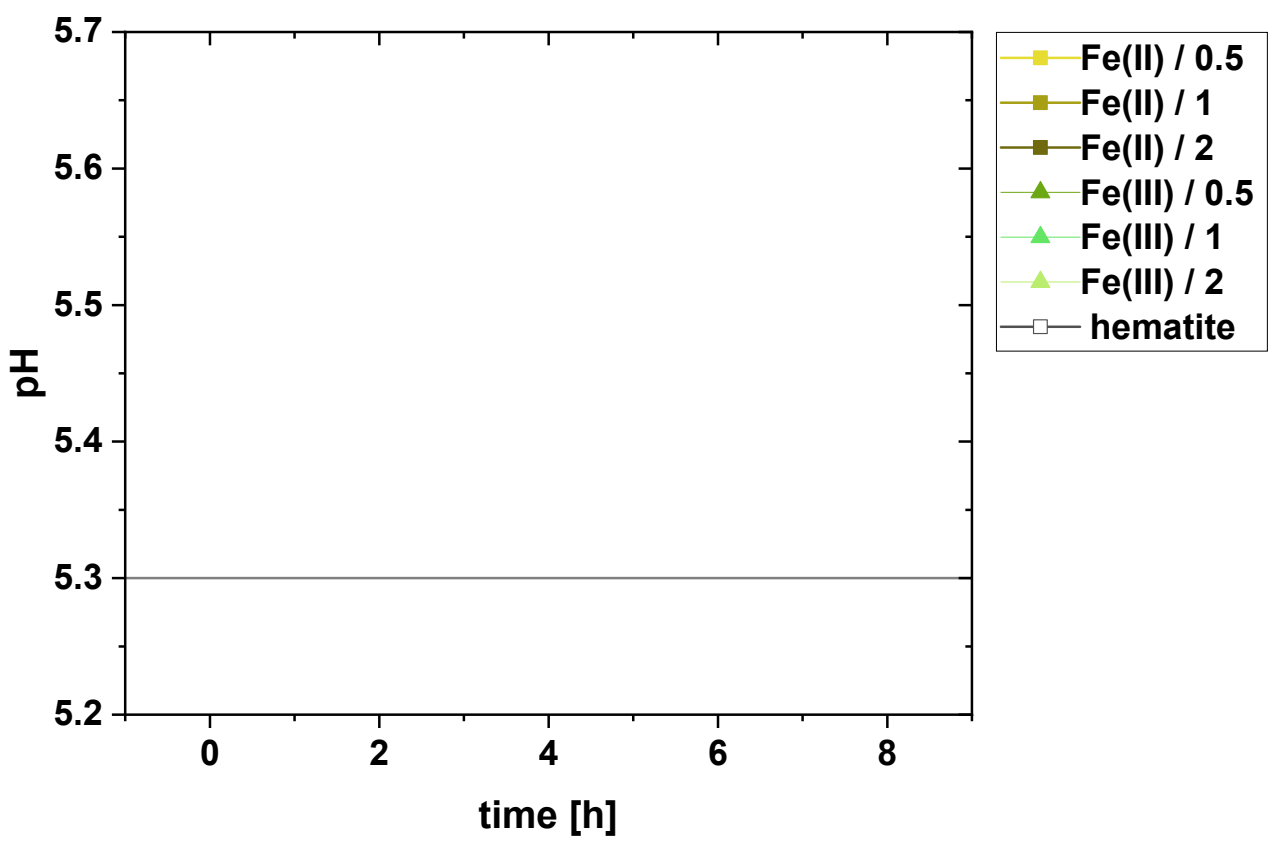

Figure S5. pH values of the reaction solutions in the 8h time period.

Table S2. Calculation of the critical nucleation barrier ΔG^* and the used parameters for the different particles. K_{sp} and ρ values were taken from (Hollemann & Wiberg, 2017; Kolthoff, 1931; Lide, 2005). The concentration used for calculation was 5 mM.

Particle	K_{sp} [mol^2/L^2]	ln(S)	v_M / N_A [10^{-29} m^3]	r^* [nm]	ΔG^* [10^{-20} J]	ΔG^* / k_BT
FeS	$4.90*10^{-18}$	29.26	3.03	0.10	0.85	2.06
$FeCO_3$	3.50*10-11	13.48	4.95	0.36	10.7	25.99
Fe_2S_3	1.40*10-88	191.69	8.06	0.04	0.14	0.34
$Fe_2(CO_3)_3$	2.79*10-39	78.18	0.13	0.16	2.01	4.90

Table S3. Theoretical Si/Fe ratio and calculated mean values Si/Fe derived from EDX measurements from multiple SEM pictures as shown in Figure S4.

Si/Fe_{theo}	$Si/Fe^{(II)}_{EDX}$	$Si/Fe^{(III)}_{EDX}$
0.5	0.35 ± 0.03	0.30 ± 0.15
1	0.61 ± 0.07	0.69 ± 0.12
2	1.06 ± 0.31	1.16 ± 0.09

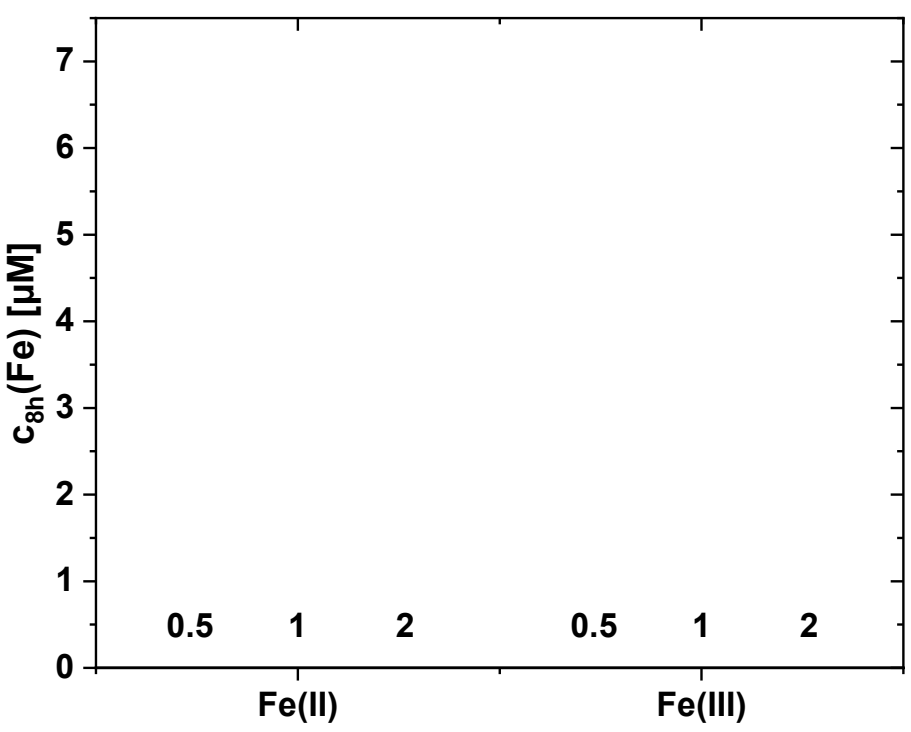

Figure S6. Leached iron concentration after 8h of reaction time.
